# Associations between Long-Term Exposure to Chemical Constituents of Fine Particulate Matter (PM_2.5_) and Mortality in Medicare Enrollees in the Eastern United States

**DOI:** 10.1289/ehp.1307549

**Published:** 2015-01-06

**Authors:** Yeonseung Chung, Francesca Dominici, Yun Wang, Brent A. Coull, Michelle L. Bell

**Affiliations:** 1 Department of Mathematical Sciences, Korea Advanced Institute of Science and Technology, Daejeon, South Korea; 2 Department of Biostatistics, Harvard T.H. Chan School of Public Health, Boston, Massachusetts, USA; 3 School of Forestry and Environmental Studies, Yale University, New Haven, Connecticut, USA

## Abstract

**Background:**

Several epidemiological studies have reported that long-term exposure to fine particulate matter (PM_2.5_) is associated with higher mortality. Evidence regarding contributions of PM_2.5_ constituents is inconclusive.

**Objectives:**

We assembled a data set of 12.5 million Medicare enrollees (≥ 65 years of age) to determine which PM_2.5_ constituents are *a*) associated with mortality controlling for previous-year PM_2.5_ total mass (main effect); and *b*) elevated in locations exhibiting stronger associations between previous-year PM_2.5_ and mortality (effect modification).

**Methods:**

For 518 PM_2.5_ monitoring locations (eastern United States, 2000–2006), we calculated monthly mortality rates, monthly long-term (previous 1-year average) PM_2.5_, and 7-year averages (2000–2006) of major PM_2.5_ constituents [elemental carbon (EC), organic carbon matter (OCM), sulfate (SO_4_^2–^), silicon (Si), nitrate (NO_3_^–^), and sodium (Na)] and community-level variables. We applied a Bayesian hierarchical model to estimate location-specific mortality rates associated with previous-year PM_2.5_ (model level 1) and identify constituents that contributed to the spatial variability of mortality, and constituents that modified associations between previous-year PM_2.5_ and mortality (model level 2), controlling for community-level confounders.

**Results:**

One–standard deviation (SD) increases in 7-year average EC, Si, and NO_3_^–^ concentrations were associated with 1.3% [95% posterior interval (PI): 0.3, 2.2], 1.4% (95% PI: 0.6, 2.4), and 1.2% (95% PI: 0.4, 2.1) increases in monthly mortality, controlling for previous-year PM_2.5_. Associations between previous-year PM_2.5_ and mortality were stronger in combination with 1-SD increases in SO_4_^2–^ and Na.

**Conclusions:**

Long-term exposures to PM_2.5_ and several constituents were associated with mortality in the elderly population of the eastern United States. Moreover, some constituents increased the association between long-term exposure to PM_2.5_ and mortality. These results provide new evidence that chemical composition can partly explain the differential toxicity of PM_2.5_.

**Citation:**

Chung Y, Dominici F, Wang Y, Coull BA, Bell ML. 2015. Associations between long-term exposure to chemical constituents of fine particulate matter (PM_2.5_) and mortality in Medicare enrollees in the eastern United States. Environ Health Perspect 123:467–474; http://dx.doi.org/10.1289/ehp.1307549

## Introduction

Regulatory control of particulate matter (PM) could be dramatically improved with robust quantification of the evidence regarding the toxicity of various constituents of the PM mixture and of their sources [U.S. Environmental Protection Agency (EPA) 2004]. Currently, PM is regulated based on the total mass concentration without regard to its chemical composition, but scientific evidence on which types of constituents are most harmful could result in more effective regulations. However, the knowledge regarding differential toxicities of PM constituents has been identified as a crucial research gap (National Research Council 2004).

Responding to the need for such evidence, for the last decade, data have been accumulated for the constituents of fine particulate matter (≤ 2.5 μm in aerodynamic diameter; PM_2.5_) nationwide in the United States and have provided opportunities for studying the association between morbidity/mortality risk and PM_2.5_ constituents. Using these data, numerous epidemiological studies have reported evidence of health effects associated with PM_2.5_ constituents, both in short-term (a few days previous) and long-term (a few years previous) exposure time frames. Studies focusing on the short-term health effects include those by Bell et al. (2014), Cao et al. (2012), Ito et al. (2011), Kim et al. (2012), Levy et al. (2012), Ostro et al. (2009), Peng et al. (2009), and Zhou et al. (2011). Fewer studies have investigated the long-term health effects of different PM_2.5_ constituents, including those by Dockery et al. (1993), Ostro et al. (2010), and Pope et al. (1995, 2002). However, studies have not reported consistent findings regarding associations with specific constituents. Such discrepancies may derive from different aspects of the study design (e.g., population, confounding control, time frame, and statistical analysis), and the U.S. EPA (2009) has called for further research.

To fill this research gap, we investigated the differential toxicity of long-term PM_2.5_ exposure according to its chemical composition, based on a large-scale national database including approximately 12.5 million Medicare enrollees (≥ 65 years of age). Combining several sources of data, we constructed a monthly multi-site time-series data set for 518 PM_2.5_ monitoring locations in the eastern region of the United States during 2000–2006. The data include, for each monitoring location, monthly mortality rates, monthly values of the average PM_2.5_ concentration over the previous 12 months, 7-year average concentrations of PM_2.5_ constituents, and community-level confounding variables on socioeconomic status (SES) and racial composition. Using a Bayesian hierarchical (BH) regression model, we estimated spatially varying (SV) mortality rates associated with previous-year PM_2.5_ and identified chemical constituents that explained the spatial variability of the mortality rates, controlling for PM_2.5_ and community-level characteristics.

## Methods

**Figure 1:**
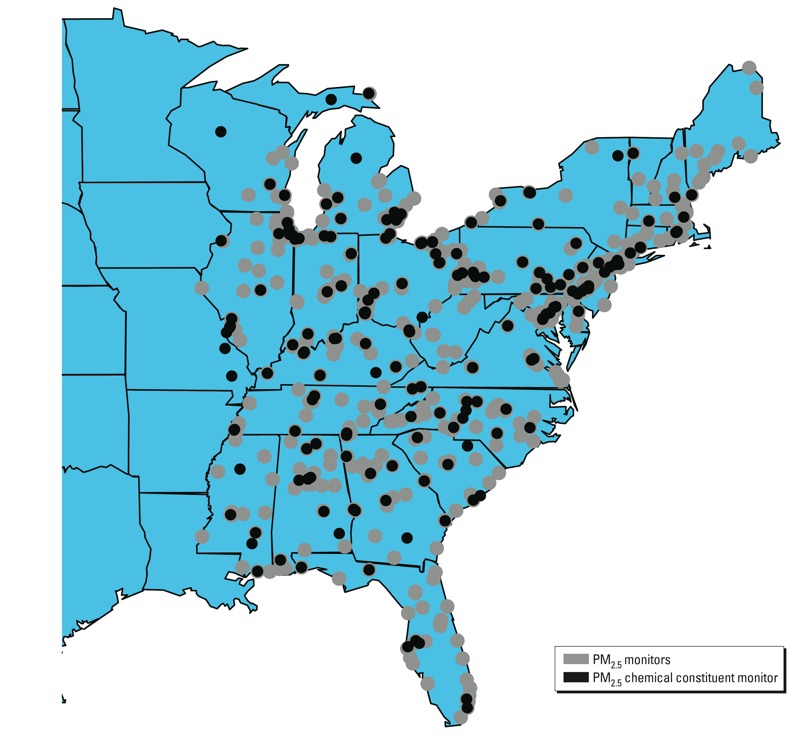
Map of 518 PM_2.5_ monitor locations and 174 PM_2.5_ chemical constituent monitor locations in the eastern region of the United States.

*Data description*. PM_2.5_ total mass. We obtained daily (24-hr average) concentrations of PM_2.5_ at 518 monitors in the eastern United States (Figure 1) for 2000–2006 from the U.S. EPA Air Quality System (AQS) database (U.S. EPA 2014). Using the daily PM_2.5_ data, we calculated monthly long-term exposure to PM_2.5_ as described in detail by Greven et al. (2011). In brief, for the first day of every month and at each of the 518 monitor locations, we calculated the previous 1-year average of daily PM_2.5_ concentrations (x*_ij_*) for *i*th monitor at *j*th month, with *i* = 1,…, *n* and *j* = 1,…, *n_i_*. Because not all monitors had valid measurements for the entire study period, the number of monthly PM_2.5_ values at a given monitoring location (*n_i_*) varied from 33 to 70.

PM_2.5_ chemical constituents. Although the U.S. EPA measures > 50 PM_2.5_ chemical constituents, we focused on 6 identified in previous research as contributing substantially to PM_2.5_ total mass (Bell et al. 2007): elemental carbon (EC), organic carbon matter (OCM), sulfate (SO_4_^2–^), silicon (Si), nitrate (NO_3_^–^), and sodium (Na). We obtained 7-year averages of the 6 chemical constituents at 174 monitors in the eastern region for 2000–2006 from the U.S. EPA AQS database, as described in detail elsewhere (Bell et al. 2007).

One challenge in our study is that the PM_2.5_ constituents are measured at monitors (174 monitors) that are not collocated with the PM_2.5_ monitors (518 monitors) (Figure 1). We assumed that the levels of constituents are spatially homogenous within a 6-mile radius, and therefore linked PM_2.5_ monitors to PM_2.5_ constituent monitors within 6 miles. Of the 518 PM_2.5_ monitors, 241 had PM_2.5_ constituent monitors within 6 miles, and we assigned 7-year averages of each PM_2.5_ constituent of the closest constituent monitor to each of 241 PM_2.5_ monitors. For the remaining 277 monitoring locations, we treated the levels of PM_2.5_ chemical constituents as missing and applied a statistical approach to impute the missing data, as described in “Statistical methods.” For either measured or imputed values, we let z*_i_* = (z*_i_*_1_,…z*_i_*_6_)´ denote the 7-year average concentrations of the six chemical constituents for *i*th PM_2.5_ monitor location.

Mortality count and total number of people at risk. Mortality counts and the total number of people at risk were obtained at ZIP-code level from billing claims of Medicare enrollees who are fee-for-service Medicare beneficiaries (≥ 65 years of age) (Greven et al. 2011). For each of the 518 PM_2.5_ monitor locations, we calculated monthly numbers of deaths and people at risk among the Medicare enrollees residing in each ZIP code with a centroid < 6 miles from a PM_2.5_ monitor location. Depending on the location, 6-mile buffers around the monitors included the centroids of at least 3 and up to 20 different ZIP codes, and the data were aggregated over 3–20 ZIP codes. We let *Y_ij_* and *N_ij_* denote the number of deaths and the people at risk for *i*th monitor location at *j*th month. For the whole study period across all 518 locations, the total size of the study population was 12.5 million enrollees, with the total number of deaths equal to 2.2 million approximately residing in 4,974 ZIP codes. For the 241 locations with PM_2.5_ constituent data available, 1.2 million deaths occurred among 7.5 million enrollees approximately living in 3,425 ZIP codes.

Community-level confounders. We obtained ZIP code–level data on community-level confounding variables including SES and racial composition from the U.S. Census 2000 (U.S. Census Bureau 2000). We averaged values over all ZIP codes with centroids within 6 miles of each PM_2.5_ monitor and assigned the averaged value to each monitor. We let w*_i_* = (w*_i_*_1_,…w*_i_*_5_)´ denote the five community-level confounders: median family income, proportion of people with high-school diploma or equivalent, proportion of residents in urban environment, proportion of white residents, and proportion of black residents.

*Statistical methods*. We analyzed the linked data using a BH Poisson regression model. The first level, a Poisson regression model with SV random effects, was used to estimate the association between month-to-month variation in mortality rate and month-to-month variation in long-term (previous 1-year average) PM_2.5_:

*Y_ij_* ~ Poisson(λ*_ij_*), *i* = 1,…, *n* and *j* = 1,…, *n_i_*,Log(λ*_ij_*) = log(*N_ij_*) + α*_i_*_0_ + α*_i_*_1_x_ij_*, [1]

where *Y_ij_* and *N_ij_* are the number of deaths and the size of the population at risk for the *i*th monitoring location and *j*th month, x_ij_* is the previous-year average PM_2.5_ centered around the location-specific average (i.e., x_ij_* _=_ x_ij_ – ^–^x_i_), and α*_i_*_0_ and α*_i_*_1_ are the location-specific (SV) random intercepts and slopes. The parameter α*_i_*_0_ represents the SV baseline mortality rate when the previous-year average PM_2.5_ is equal to its location-specific average (i.e., x_ij_* = 0). The parameter α_i1_ represents the SV association between month-to-month variation in mortality rate and month-to-month variation in previous-year PM_2.5._

The second level of the BH model regresses the location-specific 7-year averages of PM_2.5_ constituents and community-level confounders on the SV intercept and slope, α*_i_*_0_ and α*_i_*_1_:

α*_i_*_0_ = β_0_ + Σ^^6^^*__k__* __= 1__β_k_z_ik_* + Σ^^5^^*__l__* __= 1__β_6__+l_w_il_* + β_12_*^–^x_i_* + ε*_i_*_0_ [2]

α*_i_*_1_ = γ_0_ + Σ^^6^^*__k__* __= 1__γ_k_z_ik_* + Σ^^5^^*__l__* __= 1__γ_6__+l_w_il_* + ε*_i_*_1_, [3]

where z_ik_* is the level of the *k*th chemical constituent and w_il_* is *l*th community-level confounder at *i*th location, and ε*_i_*_0_ and ε*_i_*_1_ are random errors. We centered and scaled all explanatory variables to simplify interpretation and reduce multicollinearity. Note that *^–^x_i_* is included in the SV intercept in Equation 2 to control for total PM_2.5_ concentration when estimating the effects of constituents on the SV mortality rate (Mostofsky et al. 2012).

To account for potential residual spatial correlation in the second level, we assumed the error terms could be spatially correlated using a standard approach (Gelfand et al. 2003) (see Supplemental Material, “Accounting for residual spatial correlation”). We fit our BH model using a Monte Carlo Markov chain (MCMC) method [see Supplemental Material, “Two-stage estimation and the Markov Chain Monte Carlo (MCMC) algorithm”]. All computations were conducted using R statistical software (R Core Team 2013).

There are four sets of parameters of interest. From Equation 1, we obtained *a*) the SV (i.e., monitor-specific) baseline mortality rates when the previous-year PM_2.5_ was equal to its monitor-specific overall average (SV intercepts α*_i_*_0_ for each location *i*, expressed as deaths/month/1,000 persons); and *b*) the SV association between month-to-month variation in mortality rate and month-to-month variation in previous-year PM_2.5_ (SV slopes α*_i_*_1_ for each location *i*, expressed as the percentage increase in the mortality rate associated with a 1-μg/m^3^ increase in previous-year PM_2.5_). From Equations 2 and 3, we obtained *c*) the association between the SV intercepts and the monitor-specific 7-year averages of PM_2.5_ constituents, adjusted by community-level confounders and previous-year PM_2.5_ (the β*_k_* coefficients from Equation 2, expressed as the percentage increase in the mortality rate associated with a 1-SD increase in the 7-year average concentration of each constituent), and *d*) the association between the SV slopes and the monitor-specific 7-year averages of PM_2.5_ constituents, adjusted by community-level confounders (the γ*_k_* coefficients from Equation 3, expressed as the percentage increase in the mortality rate ratio for previous year PM_2.5_ associated with a 1-SD increase in the 7-year average concentration of each constituent).

To find the best fit for Equation 2, we conducted an extensive sensitivity analysis. We considered the following eight models: no explanatory variable, constituents only, community-level confounders only, or both constituents and community-level confounders as explanatory variables, all with and without spatially correlated errors. Among the eight options, we chose the best fit based on the Deviance Information Criteria (DIC) (Spiegelhalter et al. 2002).

There were 277 PM_2.5_ monitoring locations with missing values for the constituents. Separately for each constituent, we fit a Bayesian spatial Gaussian process (GP) model based on the observed data (i.e., 241 locations) and estimated a spatial correlation using the spBayes R package (Finley et al. 2007) and imputed the missing values based on the posterior predictive sample means for the 277 PM_2.5_ monitors (see Supplemental Material, “Bayesian spatial Gaussian process (GP) for missing imputation”). Before using the imputed constituent levels in the analysis, we confirmed that the Bayesian spatial GP modeling was appropriate for imputation via a cross-validation (CV) study (see Supplemental Material, “Cross validation study”).

We conducted the analysis for the complete-case data (*n* = 241 monitoring locations with the data available for both PM_2.5_ total mass and the chemical constituents) and for the all-sites data (*n* = 518 monitoring locations using imputed values for the 277 locations without measurements for PM_2.5_ chemical constituents). Also, we analyzed the data for the entire elderly population (≥ 65 years) and stratified by two age groups (65–74 vs. ≥ 75 years).

## Results

**Figure 2:**
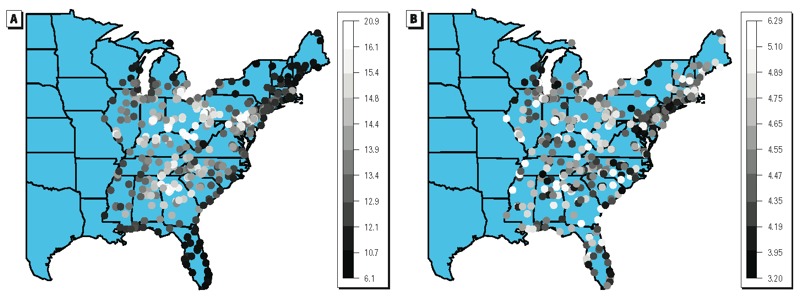
(*A*) Map of 7-year (2000–2006) averages of monthly long-term (previous 1-year average) PM_2.5_ exposure (μg/m^3^) for all PM_2.5_ monitor locations (*n* = 518). (*B*) Map of 7-year (2000–2006) averages of monthly mortality rate (deaths/month/1,000 persons) for all PM_2.5_ monitor locations (*n* = 518).

**Figure 3:**
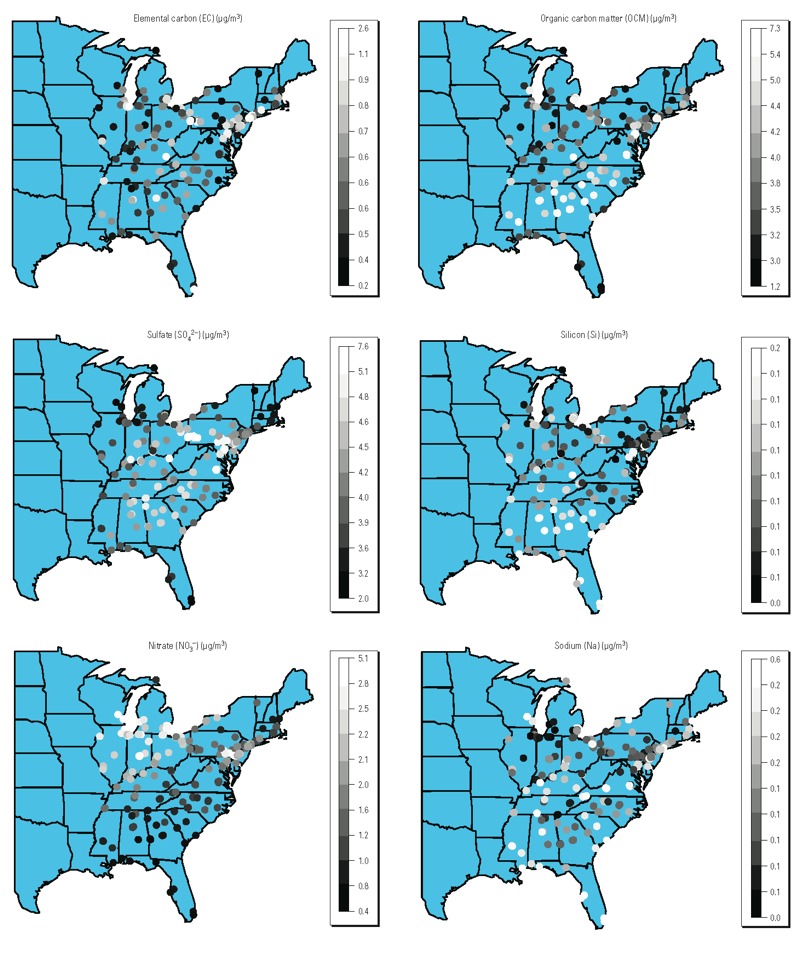
Maps of 7-year (2000–2006) averages of each of the six PM_2.5_ chemical constituents (μg/m^3^) for 241 monitor locations with available data.

Table 1 reports summary statistics for each variable for the complete-case data (*n* = 241) and for the all-sites data (*n* = 518). Figure 2A displays maps of 7-year averages of PM_2.5_ exposure levels (micrograms per cubic meter) and Figure 2B presents maps of 7-year averages of mortality rates (deaths/month/1,000 persons) for 518 monitoring locations. Figure 3 shows maps of 7-year averages of each chemical constituent (micrograms per cubic meter) for the 241 locations with available data. SO_4_^2–^ and NO_3_^–^ levels seem to exhibit strong spatial correlations; OCM, Si, and Na levels moderate spatial correlations; and EC levels weak spatial correlations, with high values only in a few locations. Estimated spatial correlations obtained from the spatial GP model between pairs of monitors with a distance of about 40 miles are 0.05, 0.20, 0.21, 0.19, 0.21, and 0.20 for EC, OCM, SO_4_^2–^, Si, NO_3_^–^, and Na, respectively. All five community-level confounders are also spatially mapped (see Supplemental Material, Figure S1) over the 518 locations.

**Table 1:** Summary statistics for each variable (mean ± SD).

Variable	Complete-case data (*n* = 241)	All-sites data (*n* = 518)
Population size at risk (*n*/month)	16901.00 ± 17307.31	14538.92 ± 15453.71
Mortality count (*n*/month)	76.01 ± 74.04	64.93 ± 66.7
Long-term PM_2.5_ exposure level (μg/m^3^)	14.56 ± 1.88	13.7 ± 2.13
PM_2.5_ chemical constituents (μg/m^3^)
Elemental carbon (EC)	0.71 ± 0.33	0.68 ± 0.24^*a*^
Organic carbon matter (OCM)	4.1 ± 1.06	4.05 ± 0.90^*a*^
Sulfate (SO_4_^2–^)	4.22 ± 0.81	4.14 ± 0.80^*a*^
Silicon (Si)	0.09 ± 0.03	0.09 ± 0.03^*a*^
Nitrate (NO_3_^–^)	1.86 ± 0.86	1.68 ± 0.85^*a*^
Sodium (Na)	0.16 ± 0.08	0.17 ± 0.07^*a*^
Community-level confounders
Family income ($)	38247.39 ± 11149.87	40305.00 ± 12461.21
Percent high school graduate	0.78 ± 0.06	0.79 ± 0.08
Percent urban	0.93 ± 0.16	0.86 ± 0.23
Percent white	0.64 ± 0.20	0.70 ± 0.20
Percent black	0.25 ± 0.18	0.19 ± 0.17
For population size at risk, mortality count and long-term (previous 1-year average) PM_2.5_, location-specific monthly values are averaged across locations for the whole study period (2000–2006). For chemical constituents, location-specific 7-year averages are averaged across locations. For community-level confounders, location-specific values are averaged across locations. ^***a***^Numbers were calculated including the imputed PM_2.5_ constituent levels for the 277 PM_2.5_ monitoring locations with missing constituent levels.		

The correlations among the 7-year averages of PM_2.5_ total mass, the PM_2.5_ chemical constituents, and community-level confounders are summarized in Supplemental Material, Table S1, for the complete-case data (*n* = 241). We observed that PM_2.5_ is correlated positively with OCM (0.43), SO_4_^2–^ (0.61), and the proportion of black residents (0.32) and inversely with Na (–0.41). The highest positive correlations among the constituents were observed between EC and OCM (0.44), SO_4_^2–^ and OCM (0.41), and Si and OCM (0.43). Among the community-level confounders, strong positive correlations were observed as 0.62 between median family income and the proportion of people with high school diploma or equivalent, and 0.50 between the proportions of white residents and high school graduates, whereas the strongest negative correlation was –0.84 between the proportions of white and black residents. Between the constituents and the community-level confounders, the highest correlations were observed for OCM at 0.39 and for Si at 0.34 with the proportion of black residents.

Before the BH regression modeling, we conducted CV studies for our imputation method for the missing constituent levels. The sample correlation coefficients between the observed and predicted values for the test data are 0.64–0.94 for all constituents averaged over 5 CV data sets (see Supplemental Material, Table S2). The root mean square error (RMSE) for prediction for each constituent and the average RMSE over five CV data sets is about half of the sample standard deviation for all constituents (see Supplemental Material, Table S3). Scatter plots for the observed versus predicted data show that the points generally follow the reference line (meaning observed values = predicted values) (see Supplemental Material, Figure S2). Based on the CV study results, we concluded that the Bayesian spatial GP method was appropriate for imputing the missing constituents in our study.

We analyzed the complete-case data (*n* = 241) and the all-sites data (*n* = 518), separately. We fit Equations 1, 2, and 3 and Supplemental Material, Equation S1 with eight different options and chose the best fit based on the smallest DIC (see Supplemental Material, Table S4). We obtained the smallest DIC for the model including both chemical constituents and community-level confounders as explanatory variables and with spatially independent errors both for the SV intercept and slope model in complete-case data as well as in the all-sites data.

**Figure 4:**
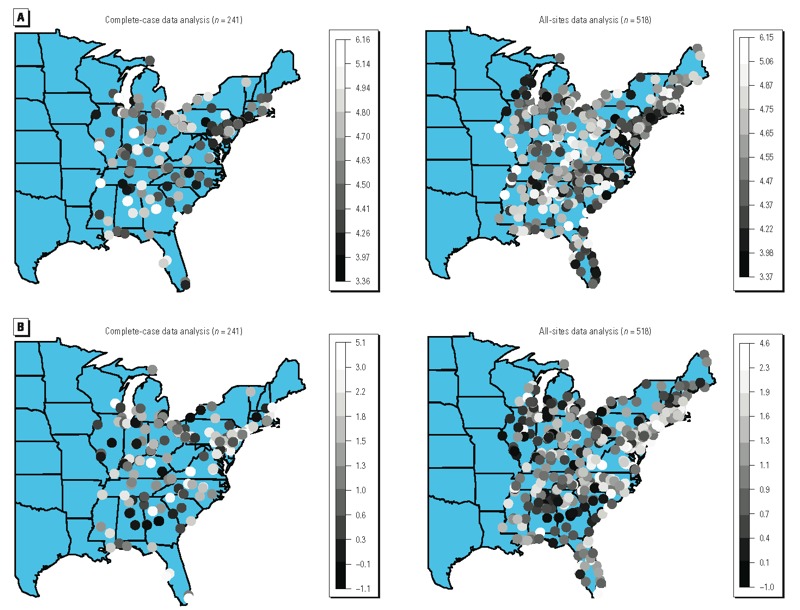
(*A*) Maps of the estimates (posterior means) of the SV intercept from the complete-case data analysis (*n* = 241, left) and the all-sites data analysis (*n* = 518, right). The values represent the monthly mortality rate (deaths/month/1,000 persons) when previous-year PM_2.5_ is at location-specific average. (*B*) Maps of the estimates (posterior means) of the SV slope from the complete-case data analysis (*n* = 241, left) and the all-sites data analysis (*n* = 518, right). The values represent the percent increase in the monthly mortality rate associated with a 1-μg/m^3^ increase in previous-year PM_2.5_.

Figure 4 displays results from the first level of the BH model for complete-case data (left panels) and all-sites data (right panels), respectively. Both sets of data showed similar results. Figure 4A shows the estimated monthly mortality rate when the previous-year PM_2.5_ is equal to its monitor-specific overall average ranges from 3.37 to 6.15 (deaths/month/1,000 persons) over the study region from all-sites data analysis. Also, Figure 4B shows that the estimated association of mortality rate with a 1-μg/m^3^ increase in the previous-year PM_2.5_ is from –1.0 to 4.6 (percent increase in mortality rate).

**Figure 5:**
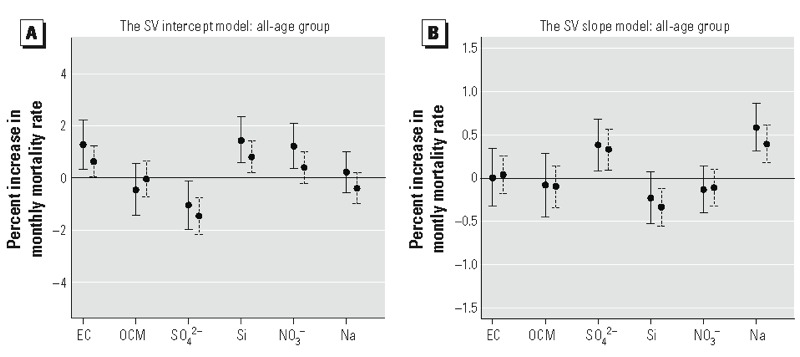
(*A*) Posterior estimates with 95% posterior intervals for the β*_k_* regression coefficients in the second-level SV intercept model. Solid error bars are for the complete-case data (*n* = 241) and dashed error bars are for the all-sites data (*n* = 518). Values correspond to the estimated percentage increase in monthly mortality rate associated with a 1-SD increase in each constituent, adjusted for previous-year average of PM_2.5_ total mass and for community-level covariates. (*B*) Posterior estimates with 95% posterior intervals for the γ*_k_* regression coefficients in the second-level SV slope model. Solid error bars are for the complete-case data (*n* = 241) and dashed error bars are for the all-sites data (*n* = 518). Values correspond to the estimated percentage increase in the association between previous-year average of PM_2.5_ and mortality when combined with a 1-SD increase in each constituent, adjusted for community level covariates.

Figure 5 reports the results from the second level of the BH model for complete-case data (left-solid bars) and all-sites data (right-dashed bars), respectively. Results were similar between complete-case data and all-sites data, but the all-sites estimates were somewhat smaller and their confidence intervals are narrower. In both analyses, we observed that adjusting for the community-level confounders and PM_2.5_ total mass, EC, Si, and NO_3_^–^ were positively associated with mortality rate (the SV intercept, β*_k_*), whereas SO_4_^2–^ was inversely related to mortality (Figure 5A). Meanwhile, SV slope estimates (γ*_k_*) indicated that the percentage increase in mortality rate with a 1-unit increase in average previous-year PM_2.5_ was greater than expected when combined with a 1-SD increase in SO_4_^2–^ and Na (Figure 5B).

Finally, we analyzed the data stratified by two age groups (65–74 vs. ≥ 75 years). For the SV intercept model (see Supplemental Material, Figure S3), results for the younger age group were similar to those for the all-age analysis (i.e., main effect estimates for EC, Si, and NO_3_^–^ were significant), whereas for the SV slope model (see Supplemental Material, Figure S4), both age groups (65–74 and ≥ 75 years) had results similar to those for the all-age analysis (≥ 65 years).

## Discussion

We investigated *a*) whether month-to-month changes in mortality rates were associated with month-to-month changes in the previous-year average exposure to PM_2.5_, and *b*) whether 7-year average levels of PM_2.5_ chemical constituents modified this association.

For the SV intercept, EC, Si, and NO_3_^–^ were positively associated with mortality rates after adjusting for PM_2.5_ total mass and the community-level confounders. For EC, our results are consistent with those of a previous cohort study of female public school professionals in California (Ostro et al. 2010). Evidence for the toxicity of Si was also found in other studies. Ostro et al. (2010) reported that long-term exposure to Si was positively associated with pulmonary mortality. Si may serve as a surrogate for toxic constituents found in mineral dust associated with traffic (Reff et al. 2009). A previous study reported that long-term exposure to traffic or traffic-related pollutants is associated with cardiopulmonary mortality (Jerrett et al. 2005). Few studies examined associations for NO_3_^–^ with mortality in a long-term framework. Ostro et al. (2010) reported that long-term exposure to NO_3_^–^ was significant for mortality in a single-pollutant model but not in a multipollutant model. Additionally, SO_4_^2–^ was found to be inversely associated with mortality rates, which is inconsistent with previous studies where positive associations were found (Dockery et al. 1993; Ostro et al. 2010; Pope et al. 1995, 2002). Although the observed positive associations were from single-pollutant approaches, our study used a multipollutant analysis that also included adjustment for the PM_2.5_ total mass and community-level confounders. Therefore, the inverse associations that we observed should be interpreted with caution, because they may be an artifact of multicollinearity resulting from correlations between SO_4_^2–^ and other constituents, PM_2.5_ total mass, and community-level confounders.

SO_4_^2–^ and Na were significant modifiers of monitor-specific associations between previous-year average PM_2.5_ and mortality rates. Previous long-term exposure studies for PM_2.5_ constituents have reported significant positive associations of SO_4_^2–^ with all-cause mortality (Dockery et al. 1993; Pope et al. 2002) or with cardiopulmonary mortality (Ostro et al. 2010; Pope et al. 2002). Na was also a significant modifier that strengthened the association between long-term PM_2.5_ and mortality. Few studies have estimated associations between Na and health outcomes, with some showing evidence of associations with mortality (Krall et al. 2013) or hospital admission (Zanobetti et al. 2009).

Several possible mechanisms have been proposed in human subject studies linking constituents to biomarkers: systemic inflammation and oxidative stress associated with EC (Neophytou et al. 2013), altered DNA methylation related to Si (Hou et al. 2014), and inflammation related to NO_3_^–^ and SO_4_^2–^ (Wu et al. 2012). However, because of limited evidences from experimental/toxicological studies, the biological pathway through which short-term exposure to PM_2.5_ and its components affects health is still an area of active investigation, and the mechanisms for long-term exposure are less understood.

The U.S. EPA measures > 50 different chemical constituents. Analyzing all available constituents would present problems of multiple comparisons. We selected the six constituents that were previously shown to be the largest contributors to PM_2.5_ total mass and/or co-vary with PM_2.5_ total mass (Bell et al. 2007). Also, the reliability of a community-level average of PM_2.5_ constituent exposure varies by constituent. For the six constituents investigated in the present study, the average correlation of monitors in close proximity (< 5 km) ranges from 0.60 to 0.93 and for larger distances (20–50 km) ranges from 0.46 to 0.88 (Bell et al. 2011). The spatial heterogeneity of many other constituents may be larger, limiting the interpretation of community-level exposures. However, we recognize that other constituents have also been found to be associated with human health. In particular, associations of health outcomes with PM_2.5_ metal constituents that were not included in our analysis, such as aluminum, calcium, chromium, lead, manganese, nickel, titanium, vanadium, and zinc, have been reported in previous studies (Bell et al. 2014; Cavallari et al. 2008; Hsu et al. 2011; Lippmann et al. 2006; Wu et al. 2012).

One limitation of the available air pollution data is that monitors that measure PM_2.5_ total mass and monitors that measure the PM_2.5_ chemical constituents are misaligned (Figure 1). We addressed this limitation by assuming that ambient levels of PM_2.5_ constituents were homogeneous within a 6-mile radius. This spatial homogeneity assumption for air pollutants builds on previous research (Bell et al. 2011) where 6 miles (about 10 km) in radius is a reasonable buffer size for the homogeneity assumption. In Bell et al. (2011), the estimated spatial correlations between pairs of monitors with distances of 5–10 km are 0.67, 0.85, 0.95, 0.62, 0.95, and 0.59 for EC, OCM, SO_4_^2–^, Si, NO_3_^–^, and Na, respectively. However, spatial variability varies by constituent—for example, with more heterogeneity for Si or Na than for SO_4_^2–^ or NO_3_^–^—and different buffer sizes may be applied for different constituents when aligning various sources of data.

Another limitation for air pollution data is that monitors that measure PM_2.5_ total mass are much denser than monitors that measure PM_2.5_ chemical constituents (Figure 1). When we aligned the two kinds of monitors, missing data occurred for almost half of the PM_2.5_ monitors. To avoid simply removing the observations with missing values and reducing the sample size to half, we adopted a Bayesian spatial GP modeling and conducted a single value imputation for the missing data separately for each constituent. We compared the results between the complete-case data analysis and the all-sites data analysis with the imputed values. Although imputation did not change our primary conclusions, results based on the imputed data should be interpreted with caution. Specifically, using a single-value imputation does not incorporate uncertainty for prediction, and measurement error can occur for the explanatory variables in regression modeling (Gryparis et al. 2009).

In our study, the PM data are the ambient levels, which we use to approximate the actual human exposure. The ambient level of a given pollutant is not a perfect surrogate of personal exposure to that pollutant, which can induce exposure measurement error into the analysis with variations in error by constituent. In a multipollutant analysis such as the present study, this type of error may induce upward bias in regression coefficient estimates, resulting in anticonservative inference on health effects. However, several authors have shown that this type of bias barely occurs in situations in which the amount of error or the correlations among pollutants in analysis are extremely large (Schwartz and Coull 2003). Therefore, it is unlikely that differences between ambient levels and personal exposures explain the observed associations in our study.

Our analysis is based on multi-site time-series data where long-term exposure was estimated by calculating previous 1-year average of daily exposure values at each temporal point (i.e., first day of each month). However, results may be sensitive to different choices of time frames. Kim et al. (2012) reported that different lag values should be selected for the short-term effects of PM_2.5_ constituents depending on health outcomes. Shorter or longer time frames than a year could be considered for examining long-term health effects of PM_2.5_.

Our study focused on the eastern region of the United States, and our findings may not be generalizable to other areas because the characteristics of PM mixtures and populations are quite different across the United States (Bell et al. 2007), and effect modification by the chemical composition of PM_2.5_ may vary among regions. Also, we focused on the elderly population, which may be more susceptible to effects of exposure than other age groups. In our study, slight differences in results were found between two age groups (65–74 and ≥ 75 years).

To our knowledge, this is the first large-scale study (covering the eastern United States) to investigate the association between long-term exposure to PM_2.5_ and mortality rate and effect modification by the chemical constituents of PM_2.5._ Unlike previous studies of PM_2.5_ constituents, we used a BH regression approach, where PM_2.5_ constituents were modeled as potential modifiers of the main effect of PM_2.5_ on health outcomes. Despite limitations, our findings add new evidence regarding the differential toxicity of PM_2.5_ constituents and their potential influence on the long-term health effects of PM_2.5_.

## Supplemental Material

(1.5 MB) PDF
