# Supplementary material for: Associations between Long-Term Exposure to Chemical Constituents of Fine Particulate Matter (PM2.5) and Mortality in Medicare Enrollees in the Eastern United States
Source: Environ Health Perspect. 2015 Jan 6;123(5):467–74. doi: 10.1289/ehp.1307549 (PMC4421760; doi:10.1289/ehp.1307549)
Supplement: (1.5 MB) PDF [file ehp.1307549.s001.508.pdf]

Environmental Health Perspectives doi:10.1289/ehp.1307549

Supplemental Material

Associations between Long-Term Exposure to Chemical Constituents of Fine Particulate Matter ($PM_{2.5}$) and Mortality in Medicare Enrollees in the Eastern United States

Yeonseung Chung, Francesca Dominici, Yun Wang, Brent A. Coull, and Michelle L. Bell

Accounting for residual spatial correlation

To account for potential residual spatial correlation, we assumed that the error terms in [2]-[3] can be spatially correlated using standard approaches (Gelfand et al. 2003) as follows:

$$\varepsilon_{i0} \sim \mathrm{N}(0, \tau_0^{-1}),\ corr(\varepsilon_{i0}, \varepsilon_{i'0}) = \exp[-\phi_0 \times \mathrm{dist}(s_i, s_{i'})]$$

$$\varepsilon_{i1} \sim \mathrm{N}(0, \tau_1^{-1}),\ corr(\varepsilon_{i1}, \varepsilon_{i'1}) = \exp[-\phi_1 \times \mathrm{dist}(s_i, s_{i'})], \qquad [S1]$$

where τ_0^{-1} and τ_1^{-1} are marginal variances, corr(ε_i, $\varepsilon_{i'}$) denotes the correlation between ε_i and $\varepsilon_{i'}$, and dist(s_i, $s_{i'}$) is the distance metric between the ith and i'th locations, with s_i denoting the coordinate vector (latitude and longitude) for the ith location, and ϕ_0 and ϕ_1 are correlation decay parameters, with larger values indicating a more rapid decay in the spatial correlation as the distance between two locations increases.

Two-stage estimation and the Markov Chain Monte Carlo (MCMC) algorithm

We conduct a Bayesian inference assuming the following priors for the unknown parameters: $\beta = (\beta_0, \dots, \beta_{12})' \sim \mathrm{MVN}(\mu_\beta, \Sigma_\beta)$ in model [2]; $\gamma = (\gamma_0, \dots, \gamma_{11})' \sim \mathrm{MVN}(\mu_\gamma, \Sigma_\gamma)$ in model [3]; $\tau_0 \sim \mathrm{Gamma}(a_0, b_0)$, $\tau_1 \sim \mathrm{Gamma}(a_1, b_1)$; $\phi_0 \sim \mathrm{Uniform}(c_0, d_0)$, $\phi_1 \sim \mathrm{Uniform}(c_1, d_1)$ in model [S1]. We obtain joint posterior distributions of the parameters of interest, β, γ, α_{i0}, and α_{i1}, using a two-stage estimation method. At the first stage, for each $PM_{2.5}$ monitoring location, we fit the regression in [1] using a maximum likelihood approach and obtain estimates of $\hat{\alpha}_{i0}$ and $\hat{\alpha}_{i1}$, and their corresponding standard errors $\hat{v}_{i0}$ and $\hat{v}_{i1}$. Then, at the second stage, we assume that $\hat{\alpha}_{i0}$ and $\hat{\alpha}_{i1}$ follow normal distributions with means equal to their true values α_{i0} and α_{i1}, and variances equal to the squared standard errors of the estimates. Then α_{i0} and α_{i1} are assumed to follow [2] and [3], respectively, with errors assumed to follow [S1], and the priors indicated above. Therefore, because of the normal likelihood, we can drive the full conditionals for all

random components (including the parameters of our interest such as β, γ, α_{i0}, and α_{i1}) and implement the Gibbs algorithm for the MCMC posterior sampling.

Bayesian spatial Gaussian process (GP) for missing imputation

For each chemical constituent k, we assume that

$$(z_{1k},\ldots, z_{nk})' = (z'_{obs,k}, z'_{miss,k})' \sim \mathrm{GP}[\mu_k 1_n,\ \psi_k^{-1} R(\phi_{zk})], \qquad \text{[S2]}$$

where $z'_{obs,k}$ and $z'_{miss,k}$ are the vectors of the observed and missing measurements, GP denotes Gaussian process, μ_k is a global mean, ψ_k is a marginal variance, and $R(\phi_{zk})$ is a spatial correlation matrix parameterized by ϕ_{zk} for kth chemical constituent. We assume exponential correlations as $\mathrm{corr}(z_{ik}, z_{i'k}) = \exp[-\phi_{zk} \times \mathrm{dist}(s_i, s_{i'})]$, with the same distance metric used in [S1].

Then, [S2] can be rewritten as

$$(z_{1k},\ldots, z_{nk})' = (z'_{obs,k}, z'_{miss,k})' \sim \mathrm{MVN}[\mu_k 1_n,\ \psi_k^{-1} R(\phi_{zk})], \qquad \text{[S3]}$$

and that the conditional distribution for $z'_{miss,k}$ given $z'_{obs,k}$ is

$$z'_{miss,k} \mid z'_{obs,k} \sim \mathrm{MVN}[\theta_{miss,k}(\mu_k, \psi_k^{-1}, \phi_{zk}), \Sigma_{miss,k}(\mu_k, \psi_k^{-1}, \phi_{zk})], \qquad \text{[S4]}$$

where the conditional mean and the conditional covariance matrix are the functions of the model parameters in [S2] and the observed measurements. Using the spBayes R package (Finley et al., 2007), we fit model [S2] for each constituent separately based on the observed data with non-informative priors and obtained the posterior predictive samples for $z'_{miss,k} \mid z'_{obs,k}$. Using the posterior predictive mean $\mathrm{E}(z'_{miss,k} \mid z'_{obs,k})$, we imputed the missing chemical constituent levels.

Cross validation study

We performed a cross validation study to confirm that the Bayesian spatial GP modeling was appropriate for imputing missing constituent concentrations using complete case data for the 241 locations. Test data were 49 randomly selected locations from the 241 with observed data, and training data were the remaining 192 locations (i.e., 20% for test and 80% for training of the

complete case data). We repeated dividing the dataset randomly 5 times to generate 5 cross validation (CV) datasets of test (missing) and training (observed) data. For each CV dataset and each constituent, we fit model [S2] and predicted the constituent concentrations for the test data based on the model fit. To evaluate prediction performance, we calculated sample correlation coefficients between observed and predicted values and the Root Mean Squared Error (RMSE) of prediction for the test data. The RMSE for *k*th constituent is defined as

$$\mathrm{RMSE_k} = \sqrt{\sum_{i=1}^{49} (z_{ik,ob} - z_{ik,pred})^2} \;, \qquad [5]$$

where $z_{ik,obs}$ and $z_{ik,pred}$ are the observed and predicted levels for *k*th constituent for *i*th location in the test data. See Tables S2 and S3 for the sample correlation coefficients and RMSE for each CV set and each constituent, and Figure S2 for scatter plots of the observed versus predicted values for each CV set and each constituent.

Table S1. Correlations among 7-year average of monthly long-term (previous 1-year average) $PM_{2.5}$, 7-year averages of $PM_{2.5}$ chemical constituents and community-level confounders using the complete case data (n=241).

	Long-term $PM_{2.5}$	EC	OCM	$SO_4^{=}$	Si	NO_3^{-}	Na	Family income	% high school graduate	% urban	% white	% black
Long-term $PM_{2.5}$	1	**0.32**	**0.43**	**0.61**	**0.09**	**0.28**	**-0.41**	**-0.15**	**-0.18**	**0.21**	**-0.23**	**0.32**
EC		1	**0.44**	**0.22**	**0.17**	**0.10**	**-0.12**	0.00	**-0.12**	**0.20**	**-0.25**	**0.14**
OCM			1	**0.41**	**0.43**	**-0.11**	-0.05	**-0.15**	**-0.23**	0.02	**-0.30**	**0.39**
$SO_4^{=}$				1	**0.23**	**-0.14**	-0.31	**-0.13**	-0.07	-0.07	0.01	**0.14**
Si					1	**-0.33**	**0.22**	**-0.21**	**-0.20**	0.00	**-0.25**	**0.34**
NO_3^{-}						1	**-0.20**	**0.18**	**0.10**	**0.24**	0.00	-0.05
Na							1	-0.03	-0.03	-0.04	-0.01	-0.02
Family income								1	**0.62**	**0.17**	**0.23**	**-0.33**
% high school graduate[a]									1	**0.25**	**0.50**	**-0.33**
% urban[b]										1	**-0.33**	**0.32**
% white[c]											1	**-0.84**
% black[d]												1

[a]The proportion of people with high school diploma or equivalent. [b]The proportion of residents in urban environment. [c]The proportion of while residents. [d]The proportion of black residents.

Note: Bold numbers are the correlation estimates that are significantly different from 0 with p-value <0.05.

Table S2. Sample correlation coefficients between observed and predicted values for the test data (n=49) for each constituent in each cross validation (CV) data set. Refer to Cross validation study for the details of the CV study.

	CV set 1	CV set 2	CV set 3	CV set 4	CV set 5	Average over 5 CV sets
EC	0.82	0.77	0.92	0.77	0.85	0.83
OCM	0.86	0.83	0.89	0.67	0.83	0.82
$SO_4^=$	0.81	0.88	0.88	0.90	0.86	0.87
Si	0.75	0.65	0.82	0.50	0.75	0.69
NO_3^-	0.92	0.96	0.97	0.94	0.91	0.94
Na	0.68	0.54	0.74	0.74	0.61	0.64

Table S3. Root mean squared error (RMSE) for prediction for the test data (n=49) for each constituent in each CV data set. Refer to Cross validation study for the details of the CV study.

	**CV set 1**	**CV set 2**	**CV set 3**	**CV set 4**	**CV set 5**	**Average over 5 CV sets**	**Sample std dev (n=241)**
EC	0.17	0.23	0.11	0.28	0.14	0.19	0.33
OCM	0.52	0.59	0.43	0.78	0.60	0.58	1.06
$SO_4^{=}$	0.39	0.35	0.29	0.34	0.33	0.34	0.81
Si	0.02	0.03	0.02	0.03	0.02	0.02	0.03
NO_3^{-}	0.25	0.21	0.17	0.33	0.33	0.26	0.86
Na	0.05	0.06	0.04	0.06	0.05	0.05	0.08

Table S4. Deviance Information Criteria (DIC) comparisons for 8 different options in equation [2] and [3] for the SV intercept and slope models.

**Explanatory variables included**	**Complete case data[a] SV intercept**	**Complete case data SV slope**	**All sites data[b] SV intercept**	**All sites data SV slope**
**Spatially correlated errors**				
No predictor	-480.988	-477.279	-1036.07	-961.690
Constituents only	-491.030	-477.568	-1046.15	-961.822
Community-level confounders only	-518.069	-479.350	-1066.68	-963.002
Constituents + Community-level confounders	-518.440	-479.630	-1071.30	-962.942
**Spatially not-correlated errors**				
No predictor	-400.494	-1207.22	-918.934	-2562.56
Constituents only	-427.690	-1215.37	-935.799	-2575.10
Community-level confounders only	-556.237	-1223.83	-1215.01	-2586.73
Constituents + Community-level confounders[c]	-580.750	-1224.56	-1227.32	-2589.53

[a]N = 241. [b]N = 518. [c]DICs from these models are the smallest values for each column.

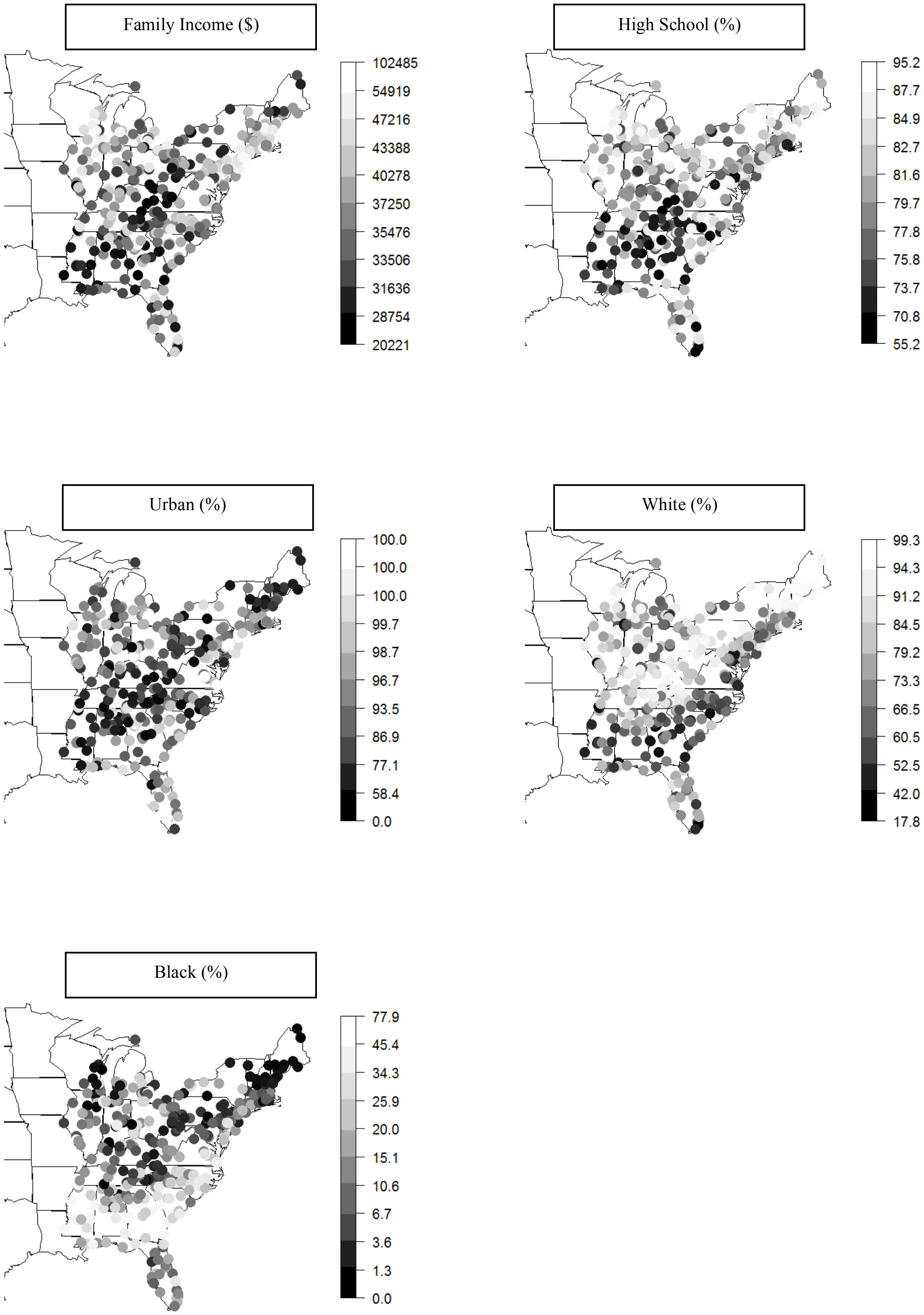

Figure S1. Maps for community-level confounders from the US Census 2000 for all $PM_{2.5}$ monitor locations (n=518).

	CV set 1	CV set 2	CV set 3	CV set 4	CV set 5
**EC**	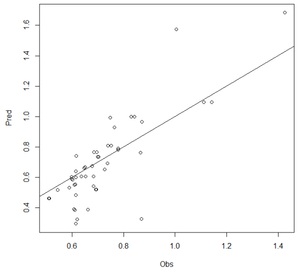	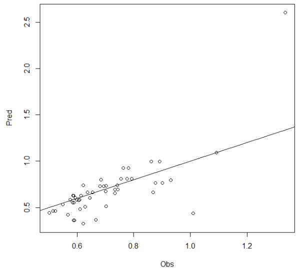	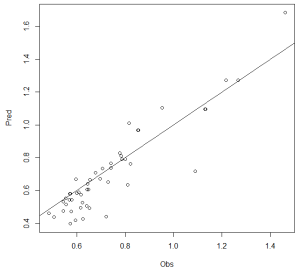	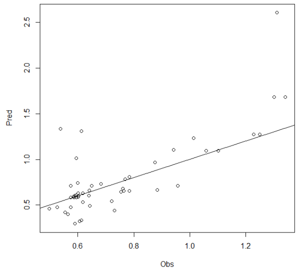	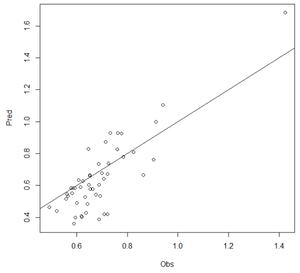
**OCM**	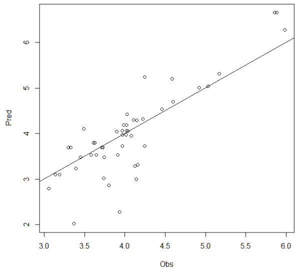	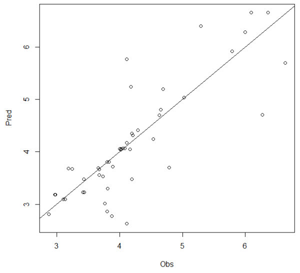	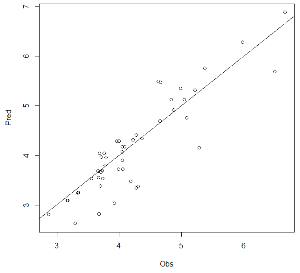	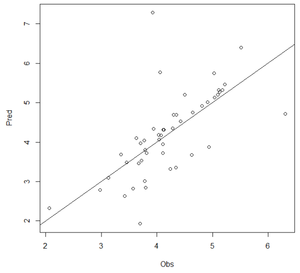	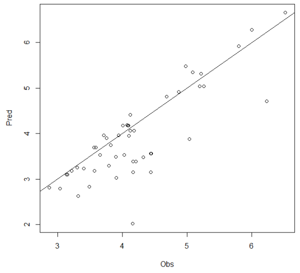
**$SO_4^=$**	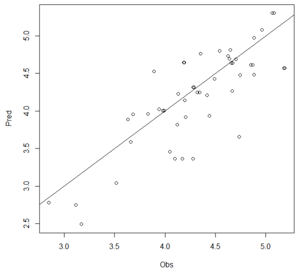	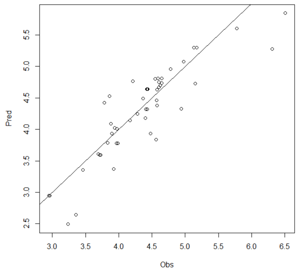	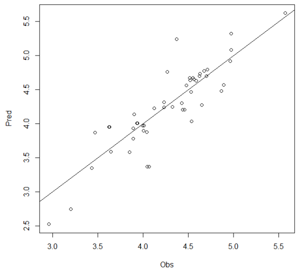	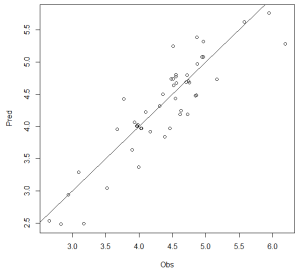	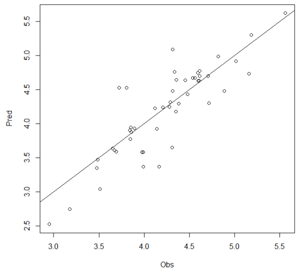

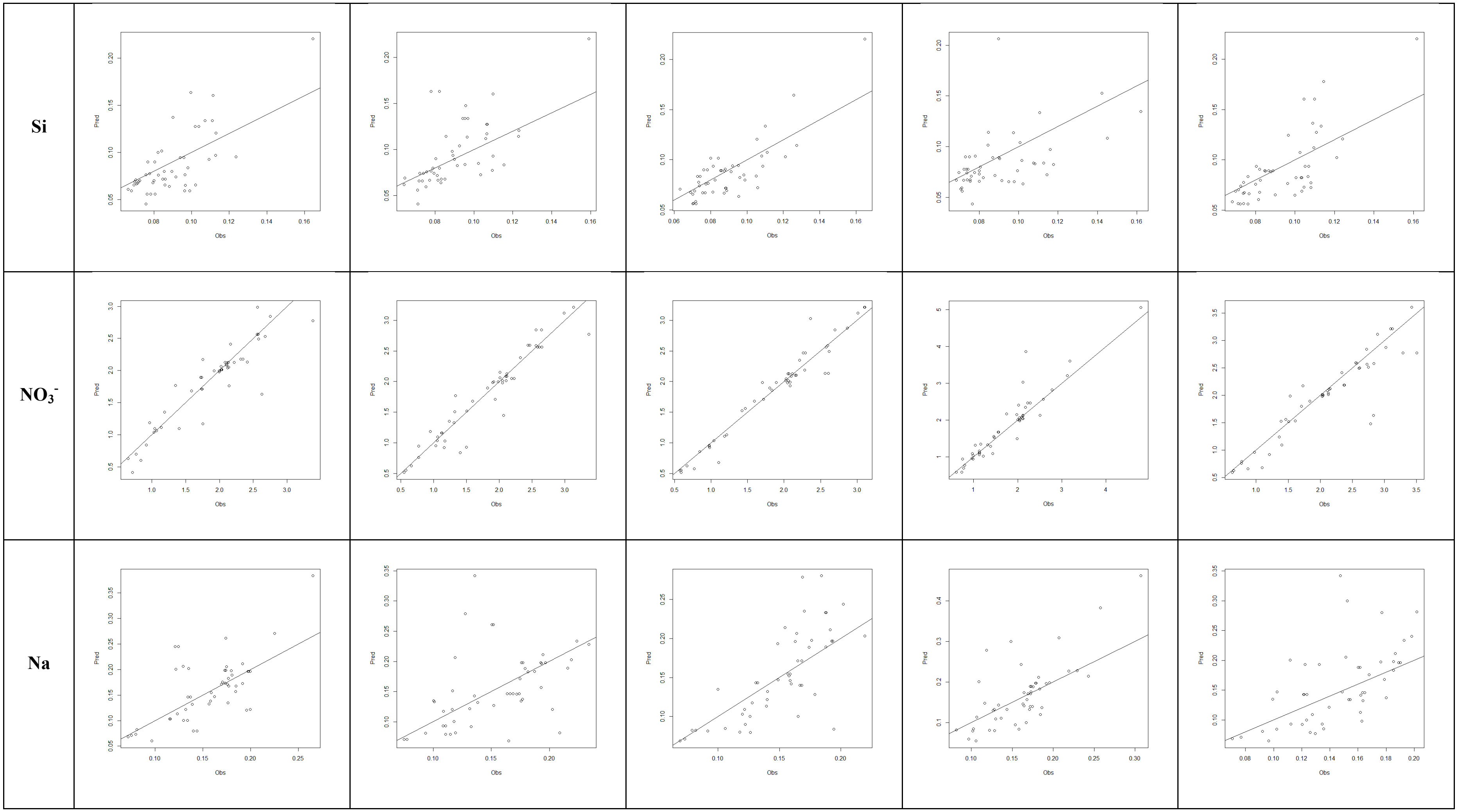

Figure S2. Scatter plots for the observed (x-axis) vs predicted (y-axis) values for the test data (n=49) for each constituent in each CV data set. Refer to Cross validation study for the details of the CV study.

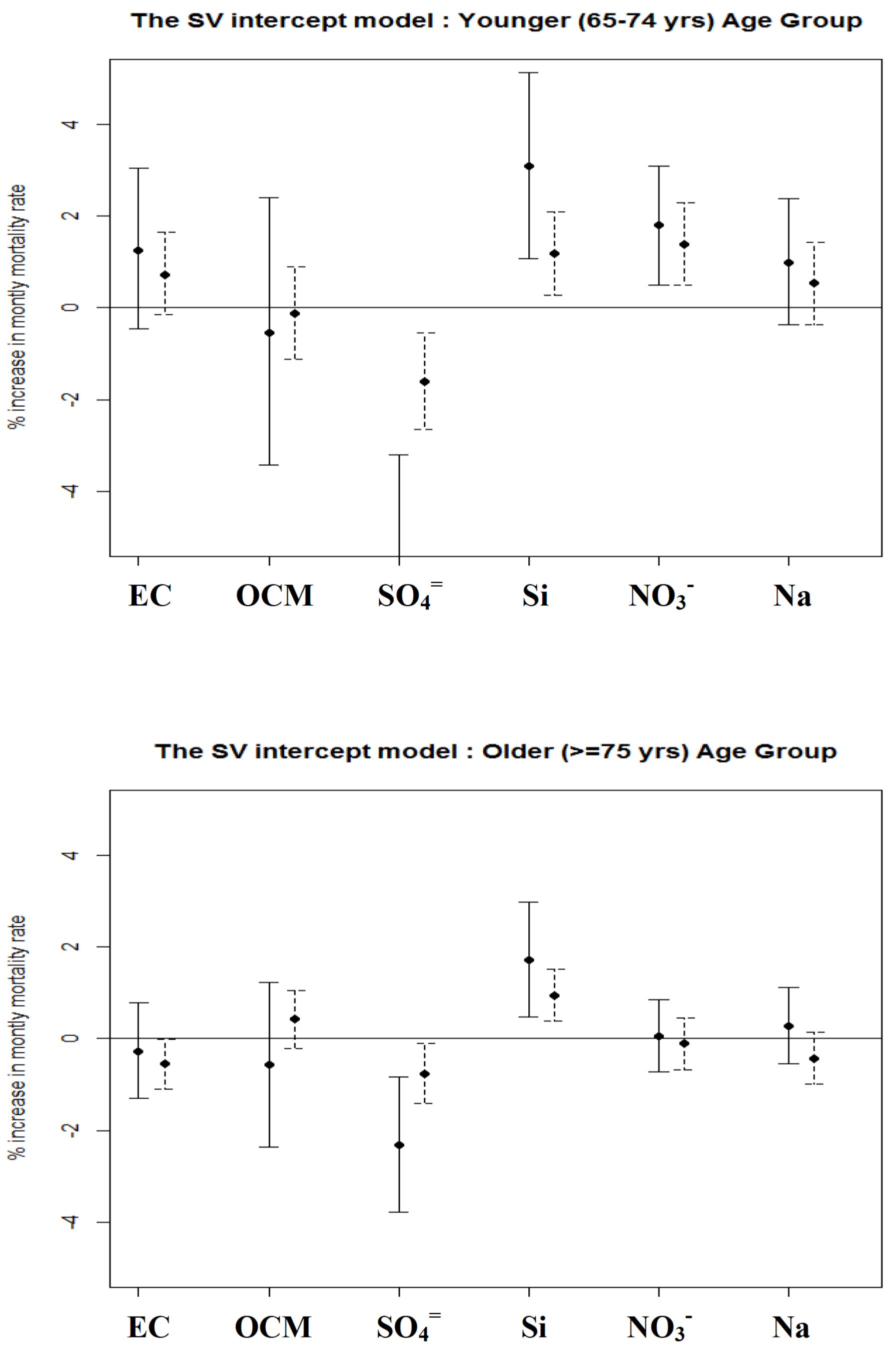

Figure S3. Posterior estimates with 95% posterior intervals for the β_k regression coefficients in the second-level SV intercept model for two age groups (65-74, ≥75 yrs). Left-solid bars are for the complete case data (n=241) and right-dashed bars are for the all sites data (n=518). Values correspond to the estimated percentage increase in monthly mortality rate associated with a 1-SD increase in each constituent, adjusted for previous-year average of $PM_{2.5}$ total mass and for community-level covariates.

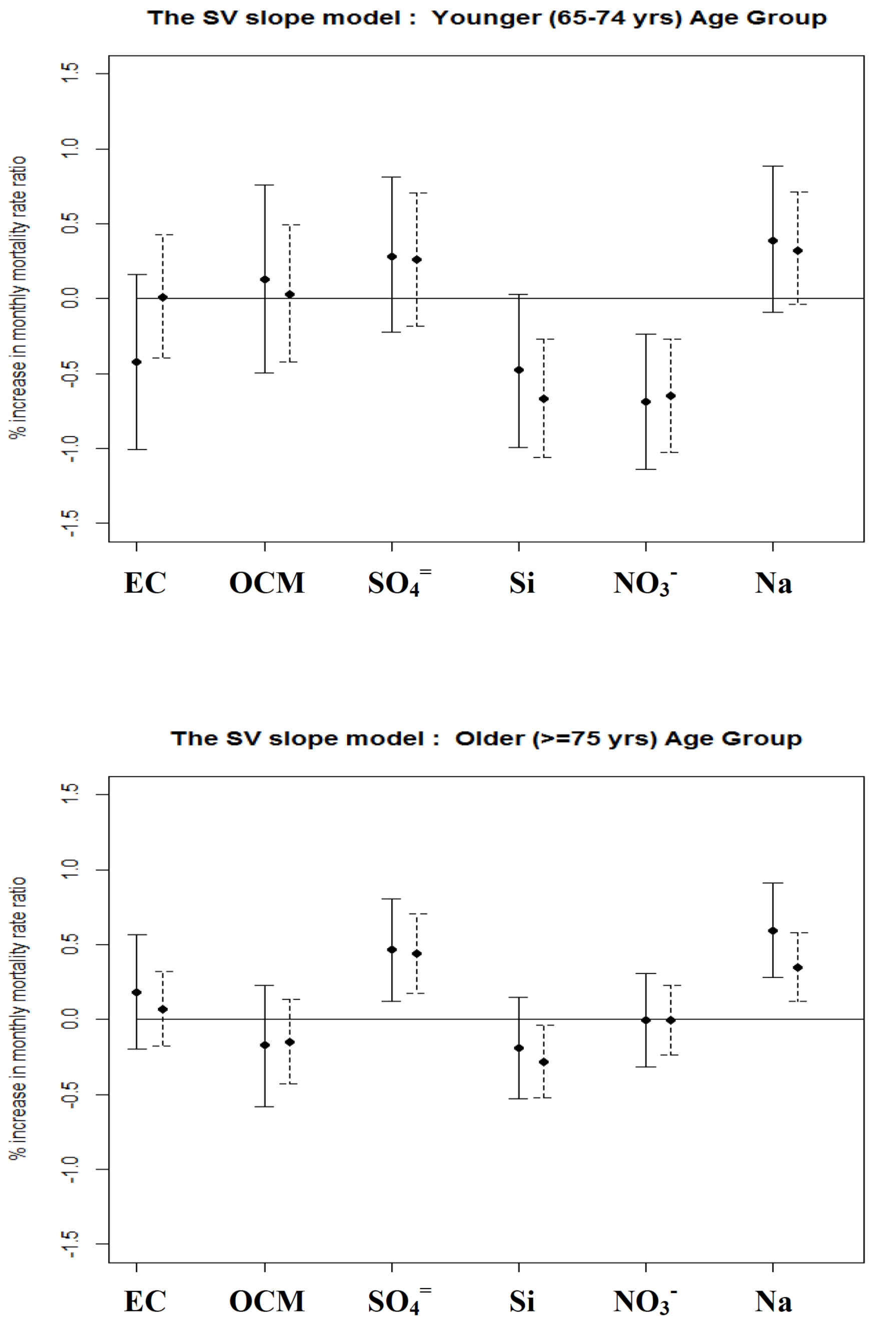

Figure S4. Posterior estimates with 95% posterior intervals for the γ_k regression coefficients in the second-level SV slope model for two age groups (65-74, ≥75 yrs). Left-solid bars are for the complete case data (n=241) and right-dashed bars are for the all sites data (n=518). Values correspond to the estimated percentage increase in the association between previous-year average of $PM_{2.5}$ and mortality when combined with a 1-SD increase in each constituent, adjusted for community level covariates.
